# Cardio Pulmonary Resuscitation 2010 - Improve the quality of care

**DOI:** 10.4103/0019-5049.63634

**Published:** 2010

**Authors:** S S Harsoor

**Affiliations:** Editor, Indian Journal of Anesthesia, No. 21, 2^nd^ Cross, Kirloskar Colony, Basaveshwar Nagar, 2^nd^ Stage, Bangalore – 560 079, India. E-mail: editor@ijaweb.org

The cardiopulmonary resuscitation or CPR is an emergency medical procedure performed in a victim of cardiac or respiratory arrest and it consists of rapid chest compressions to maintain artificial blood circulation, along with artificial respiration. It is continued either till the return of spontaneous circulation (ROSC) or till the person is declared dead. The purpose of CPR is to maintain the circulation to brain and heart, thereby postpone the tissue death and brain damage, until cardiac activity is restarted by advanced cardiac life support (ACLS), commonly defibrillation. The American Heart Association (AHA) and International Liaison Committee on Resuscitation (ILCOR) have laid down the CPR guidelines in 2005 which are modified every 5 years and the next modification is due in late 2010.

It is described in The Bible, wherein similarity to CPR is mentioned in the pages of Books of King's (II 4:34), wherein Prophet Elisa warms the dead boy's body and "places his mouth over his…"

Artificial respiration is also attempted by Silvester (The Silvester method) where arm is used to aid respiration. Holger Neilson technique (1911) is also a similar technique to aid respiration. Mouth-to-mouth (MTM) respiration was added on the assumption that it will oxygenate the blood and its combination with chest compression is more effective for CPR.

AHA guidelines for CPR and Emergency Cardiovascular Care (ECC) say: "*Laypersons should be encouraged to do compression-only CPR if they are unable or unwilling to provide rescue breaths although the best method of CPR is compressions coordinated with ventilations*."[1] The statement contained a secondary conclusion that "*...provision of chest compression without mouth-to-mouth ventilation is far better than not attempting resuscitation at all*." Reasons cited prospectively for the reluctance to perform CPR included concerns about disease transmission related to performing MTM ventilation.

It is emphasized that chain of survival depends on the following factors.

1. Early CPR to minimize organ injury and buy time.
2. Early defibrillation to restore circulation.
3. Early advanced life support and post resuscitation care to restore quality of life by enhancing the recovery of neurological function.

The ILCOR, a body of seven international resuscitation organizations (AHA, ERC, IAHF, HSFC, ANZCOR, RCSA, and RCA), published the 2005 CPR guidelines[2] with a goal of simplifying CPR for lay rescuers and healthcare providers alike and to maximize the potential for early resuscitation. The guidelines include

1. a universal compression:ventilation ratio (30:2), instead of 15:2 (except in infants),
2. removal of the emphasis on lay rescuers assessing for pulse or signs of circulation for an unresponsive adult victim, and
3. taking the absence of breathing as the key indicator for starting CPR rather than an absent pulse, in an unresponsive victim, since it is observed that lay persons could detect pulse in only 40% of victims.

Following a sudden cardiac arrest, three distinct physiologic phases in the body, especially in heart and brain, are described.

1. Electrical phase: In the first 4–5 minutes, defibrillation has most dramatic effect and it is highly successful.[3]
2. Haemodynamic phase: In the next 4–5 minutes due to circulatory failure, the fibrillating heart depletes all the myocardial high-energy phosphate bonds and later resuscitation of normal contractile activity becomes more difficult. Hence, early uninterrupted chest compressions will provide coronary and cerebral circulations and help in the attempts to defibrillate the heart and restore spontaneous circulation. Since the pressures generated by chest compressions are quite low compared to intact circulation, the interruptions are to be strongly discouraged.
3. Metabolic: CPR is only likely to be effective if commenced within 6 minutes after the blood flow stops,[3] because permanent brain cell damage occurs when fresh blood infuses the cells after this, since the cells of the brain become dormant in as little as 4–6 minutes in an oxygen-deprived environment and the cells are unable to survive the reintroduction of oxygen in a traditional resuscitation. Hypothermia seems to protect the victim by slowing down metabolic and physiologic processes, greatly decreasing the tissues' need for oxygen.

## COMPRESSION ONLY (CARDIOCEREBRAL) RESUSCITATION

The compression-only CPR, also known as cardiocerebral resuscitation (CCR) involves simply chest compressions without artificial respiration. The CCR method has claimed a 300% greater success rate over standard CPR with the exceptions of drowning or drug overdose. A Japanese study claimed strong evidence that compressing the chest, and not mouth-to-mouth (MTM) ventilation, is the key to helping a person recover from cardiac arrest.[4] Thus, on March 30, 2008, the AHA broke away from the ILCOR position and stated that compression-only CPR works as well as, and sometimes better than, traditional CPR. In a review, Ewy observed that application of CCR methods, both in rural as well as urban population, has more than 300% survival to hospital discharge.[5] But the application of CCR in children is rather ambiguous. A cohort study published observed that in children who are victims of arrest due to noncardiac causes, the conventional CPR produced better results, whereas for arrests of cardiac causes, both the conventional CPR and compression-only CPR (CCR) are equally effective.[6] But Nagao strongly recommends that compression-only CPR by a bystander is known to all, is recommended and taught because it is a preferable approach to basic life support (BLS) in adult victims.[4]

But the European Resuscitation Council, after reviewing the then available published scientific evidence, considered thus: "the current evidence is insufficient to alter its guidelines for BLS at this moment." A new consensus on science will be published in 2010 and it is appropriate to await the outcome of this process before new changes in the guidelines are recommended.[7] It is not in the interest of the quality of CPR to introduce new changes while the current guidelines are just being implemented. The resulting confusion will be counterproductive. The need to simplify guidelines, potentially at the expense of quality, just to encourage lay rescuers to perform CPR should be considered as minimal.

The European Resuscitation Council therefore continues to recommend the teaching and administration of high quality, minimally interrupted chest compressions at a rate of 100 per minute alternated with two MTM ventilations in a ratio of 30:2. For those laypersons who are unwilling or unable to give MTM ventilations, chest compression-only is much more acceptable than performing no CPR at all.

## DEFIBRILLATION

The electric current of adequate magnitude passes across the myocardium from the electrode paddles placed over the apex and base of ventricles so that the fibrillating myocardium is depolarized thus enabling the intrinsic pacemaker of the victim's heart to generate a stable perfusing rhythm. It is shown that CPR increases the success rate of defibrillation by maintaining the coronary perfusion during the interim period after cardiac arrest though this benefit exists within a narrow window of effectiveness.[3] It is recommended to use a single shock of 150–200 J for biphasic current and 360 J for monophasic current, at the earliest possible time. Since there is a possible delay in establishment of palpable pulse, CPR must be continued soon after the shock to minimize the organ damage due to ischemia.

**Table:** Time factor (CPR statistics)

Type of arrest	ROSC (%)	Survival (%)
Witnessed in-hospital cardiac arrest	48	22
Unwitnessed in-hospital cardiac arrest	21	1
Bystander CCR	40	6
Bystander CPR	40	4
No bystander CPR (ambulance CPR)	15	2
Defibrillation within 3–5 minutes	74	30

The availability and successful deployment of automated external defibrillators (AED) can certainly improve the survival of arrest victims. Tha application of AED in communities was associated with nearly doubling of survival, and Weisfeldt and others highlighted the importance of strategically expanding the community-based AED program.[8]

Certain devices use techniques such as pneumatics to drive a compressing pad on to the chest of the patient. One such device, known as the LUCAS, developed at the University Hospital of Lund, is powered by the compressed air cylinders or lines available in ambulances or in hospitals. After numerous clinical trials, it has shown a marked improvement in coronary perfusion pressure and return of spontaneous circulation.[9]

Another system called the AutoPulse is electrically powered and uses a large band around the patient's chest which contracts in rhythm in order to deliver chest compressions. This is also backed by clinical studies showing increased successful return of spontaneous circulation.[10]

The post resuscitation period is often marked by haemodynamic instability as well as laboratory abnormalities. This is also a period for which promising technological interventions such as controlled therapeutic hypothermia are being evaluated. The available experimental evidence suggests that therapeutic hypothermia is beneficial. After a systematic review, Arrich and others suggested that any conventional cooling method to induce mild therapeutic hypothermia in adult victims seems to improve the survival and neurological outcome after cardiac arrest.[11]

Every organ system is at risk during this time, and patients may ultimately develop multiorgan dysfunction.

Initial objectives of post resuscitation care are to

1. optimize cardiopulmonary function and systemic perfusion especially perfusion to the brain;
2. transport the victim of out-of-hospital cardiac arrest to the hospital emergency department (ED) and continue care in an appropriately equipped critical care unit;
3. try to identify the precipitating causes of the arrest;
4. institute measures to prevent recurrence;
5. institute measures that may improve long-term, neurologically intact survival.

AHA observes that despite four decades of promulgation, it is a serious problem that majority of the bystanders are either unwilling or unable to perform CPR, despite several studies confirming the effectiveness and survival of arrest victims[12] and a higher discharge rate following implementation of AHA guidelines.[13] When effectively trained, even the lay persons in the age group of 50–76 years were able to perform CPR with acceptable quality for more than 10 minutes. Hence, there should not be any age bar in teaching and training the laypersons in CPR skills.[14]

The mission of AHA/ILCOR is focused strictly on evidence-based scientific consensus reached by the dedicated physicians, through a comprehensive and unbiased review process, so that new CPR guidelines are published in later 2010.

The scientific presentation of any subject is constantly changing and the mission should be to translate new scientific observations into saving more lives. All age groups of persons can contribute their might to save more lives. The need of the hour is to improve the quality of care and involve and educate everyone interested to contribute to save more lives. Late 2010 will see a new era with new guidelines of CPR.

## References

[1] (2005). 2005 American Heart Association Guidelines for Cardiopulmonary Resuscitation and Emergency Cardiovascular Care (AHA), Circulation. 112, 24 Supplement.

[2] (2005). International Liaison Committee On Resuscitation. Consensus on Science and Treatment Recommendations. Resuscitation.

[3] Cummins RO, Eisenberg MS, Hallstrom AP, Litwin PE (1985). Survival of out-of-hospital cardiac arrest with early initiation of cardiopulmonary resuscitation. Am J Emerg Med.

[4] Nagao KftS-Ksg (2007). Cardiopulmonary resuscitation by bystanders with chest compression only (SOS-KANTO): an observational study. Lancet.

[5] Ewy GA (2008). Cardiocerebral resuscitation: a better approach to cardiac arrest. Curr Opin Cardiol.

[6] Kitamura T, Iwami T, Kawamura T, Nagao K, Tanaka H, Nadkarni VM (2010). Conventional and chest-compression-only cardiopulmonary resuscitation by bystanders for children who have out-of-hospital cardiac arrests: a prospective, nationwide, population-based cohort study. Lancet.

[7] (2008). Advisory statement of the European Resuscitation Council, 31 March.

[8] Weisfeldt ML, Sitlani CM, Ornato JP, Rea T, Aufderheide TP, Davis D (2010). Survival after application of automatic external defibrillators before arrival of the emergency medical system: evaluation in the resuscitation outcomes consortium population of 21 million. J Am Coll Cardiol.

[9] Rubertsson S, Karlsten R (2005). Increased cortical cerebral blood flow with LUCAS; a new device for mechanical chest compressions compared to standard external compressions during experimental cardiopulmonary resuscitation. Resuscitation.

[10] Casner M, Andersen D, Isaacs SM (2005). The impact of a new CPR assist device on rate of return of spontaneous circulation in out-of-hospital cardiac arrest. Prehosp Emerg Care.

[11] Arrich J, Holzer M, Herkner H, Müllner M (2010). Cochrane corner: hypothermia for neuroprotection in adults after cardiopulmonary resuscitation. Anesth Analg.

[12] Ong ME, Ng FS, Anushia P, Tham LP, Leong BS, Ong VY (2008). Comparison of chest compression only and standard cardiopulmonary resuscitation for out-of-hospital cardiac arrest in Singapore. Resuscitation.

[13] Aufderheide TP, Yannopoulos D, Lick CJ, Myers B, Romig LA, Stothert JC (2010). Implementing the 2005 American Heart Association Guidelines Improves Outcomes after Out-of-Hospital Cardiac Arrest. Heart Rhythm.

[14] Neset A, Birkenes TS, Myklebust H, Mykletun RJ, Odegaard S, Kramer-Johansen J (2010). A randomized trial of the capability of elderly lay persons to perform chest compression only CPR versus standard 30:2 CPR. Resuscitation.

